# Supplementary material for: Methylglyoxal-Derived Nucleoside Adducts Drive Vascular Dysfunction in a RAGE-Dependent Manner
Source: Antioxidants (Basel). 2024 Jan 10;13(1):85. doi: 10.3390/antiox13010085 (PMC10812505; doi:10.3390/antiox13010085)
Supplement: Supplementary file 1 [file antioxidants-13-00085-s001.zip › antioxidants-2788667-supplementary.pdf]

Supplemental Material

Methylglyoxal-derived nucleoside adducts drive vascular dysfunction in a RAGE-dependent manner

Seigmund Wai Tsuen Lai[1], Supriyo Bhattacharya[2], Edwin De Jesus Lopez Gonzalez[1], Sarah C. Shuck[1]*

[1]Department of Diabetes and Cancer Metabolism, Arthur Riggs Diabetes and Metabolism Research Institute, City of Hope Comprehensive Cancer Center, Duarte, CA, United States

[2]Department of Computational and Quantitative Medicine, City of Hope National Medical Center, Duarte, CA, United States

*Corresponding author: sshuck@coh.org

Table S1. Human primer sequences used for quantitative PCR.

Target	NCBI Gene Accession Number	Forward (5'→3')	Reverse (5'→3')
ICAM1	NC_000019.10	GTGTCCTGTATGGCCCCCGACT	ACCTTGCGGGTGACCTCCCC
VCAM1	NC_000001.11	GTCAATGTTGCCCCCAGAGA	TTTTCGGAGCAGGAAAGCCC
ENOS	NC_000007.14	TGATGGCGAAGCGAGTGAAG	ACTCATCCATACACAGGACCC
TSP1	NC_000015.10	AGACTCCGCATCGCAAAGG	TCACCACGTTGTTGTCAAGGG
LINC00607	NC_000002.12	ACCGGGCGTTGAGAATACAA	ACACTTGGCGAAACTTCCCT
TNFA	NC_000006.12	ATGGAGACAGATGTGGGGTGT	CTTCCAGGCATTCAACAGCTC
IL1B	NC_000002.12	ACTGGCGAGCTCAGGTACT	CCATGCACTGGATGCTGAGAG
IL6	NC_000007.14	AGCAGGCACCCCAGTTAATC	ATTTGTGGTTGGGTCAGGGG
IFNG	NC_000012.12	TGATTTCCTTTTCAACTCTTCTGCT	TGGGACCTTTGGAGTATCAGC
RAGE	NC_000006.12	TCACCCTTCTCATTAGGCAC	TACCATGGTGCTATCTCCCA
GAPDH	NC_000012.12	TTGGCTACAGCAACAGGGTG	GGGGAGATTCAGTGTGGTGG

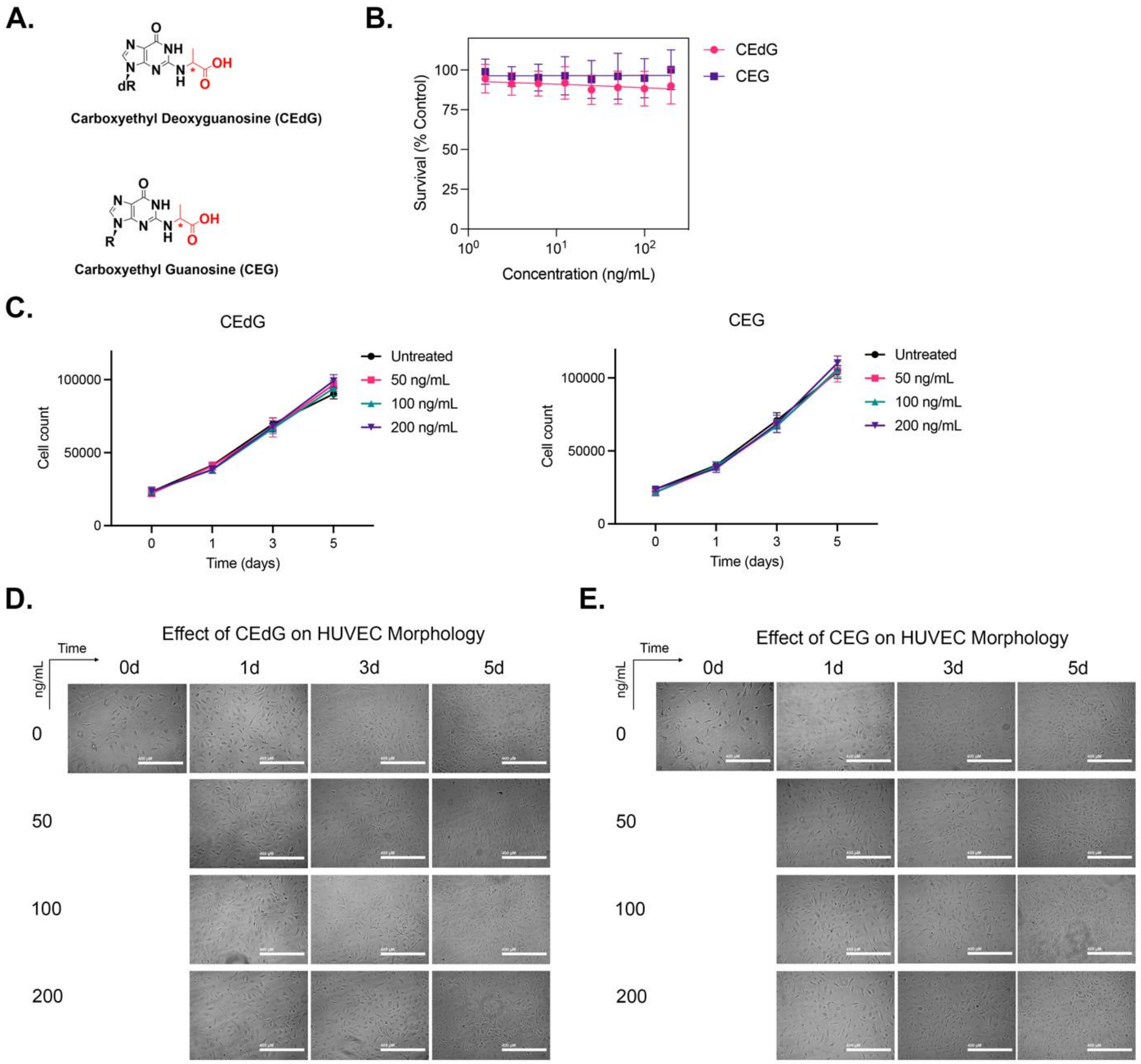

Figure S1. MG-adducts do not impact HUVEC viability, proliferation, or morphology. (A) Chemical structures of CEdG and CEG. (B) Impact of CEdG or CEG on viability in HUVECs treated for 24 hr at doses up to 200 ng/mL of CEdG or CEG. (C, D) Impact of CEdG or CEG up to 200 ng/mL on HUVEC proliferation. (E, F) Impact of CEdG or CEG up to 200 ng/mL on HUVEC morphology.

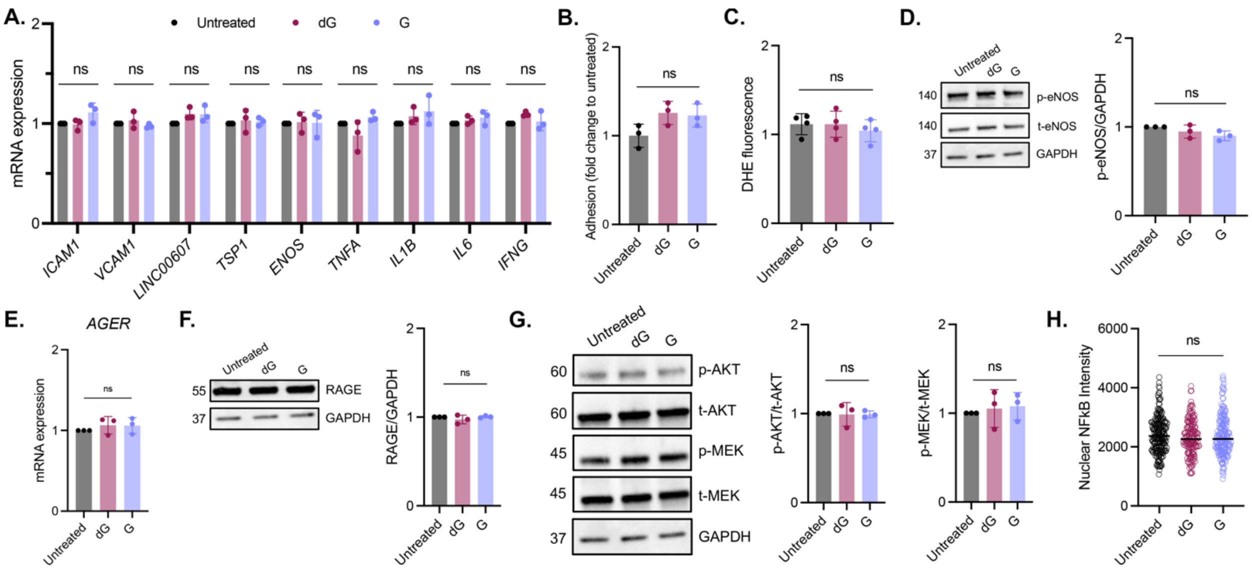

Figure S2. Unmodified nucleosides do not induce endothelial dysfunction, activate RAGE, or promote NFκB nuclear translocation. (A) qPCR analysis of *ICAM1, VCAM1, LINC00607, TSP1, ENOS, TNFA, IL1B, IL6,* and *IFNG* in HUVECs treated for 1 hour with 100 ng/mL of dG or G. (B) Functional effects on endothelial activation was assessed via monocyte adhesion assay. HUVECs were treated with 100 ng/mL of dG or G for 1 hr. (C) ROS were detected via dihydroxyethidium (DHE) probe in HUVECs treated with 100 ng/mL dG or G for 1 hr. (D) Western blot analysis for phosphorylated and total eNOS in HUVECs treated with 100 ng/mL of dG or G for 1 hr. (E) qPCR analysis of *AGER* expression in HUVECs treated with 100 ng/mL of dG or G for 1 hr. (F) Western blot analysis of RAGE expression in HUVECs treated with 100 ng/mL of dG or G for 1 hr. (G) Western blot analysis of RAGE activation in HUVECs treated with 100 ng/mL of dG or G for 1 hr. (H) NFκB nuclear translocation was measured via immunofluorescence and brightfield microscopy. HUVECs were treated with 100 ng/mL dG or G for 1 hr.

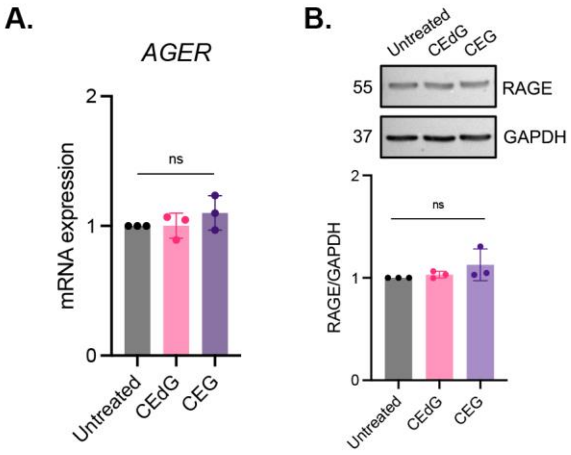

Figure S3. MG-adducts do not impact RAGE gene and protein expression in HUVECs. (A) qPCR analysis of *AGER* expression in HUVECs treated with 100 ng/mL of *R, S*-CEdG or CEG for 1 hr. (B) Western blot analysis of RAGE expression in HUVECs treated with 100 ng/mL of *R, S*-CEdG or CEG for 1 hr.

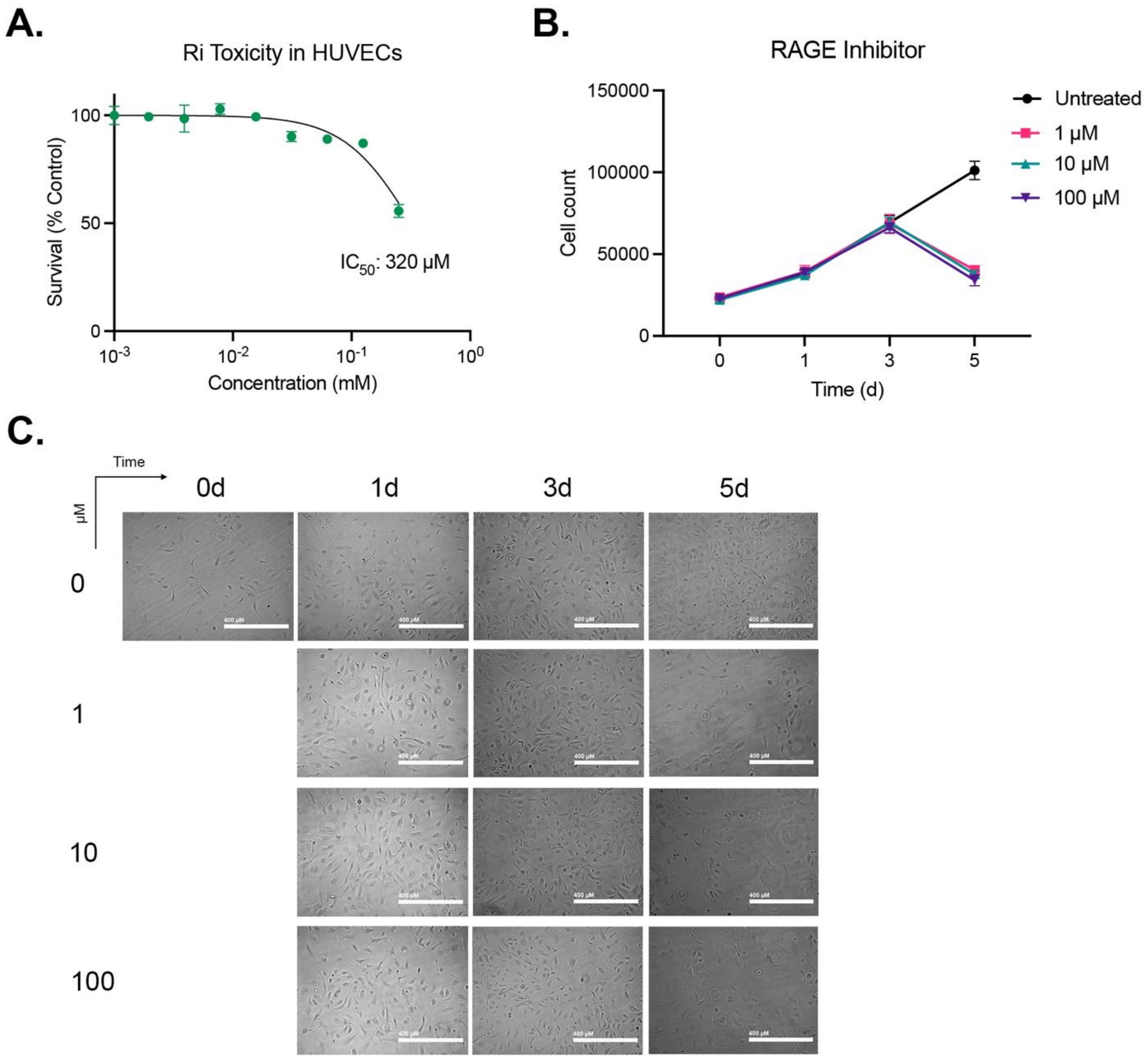

Figure S4. Impact of Ri on HUVEC viability, proliferation, and morphology. (A) HUVEC viability measured using crystal violet staining following treatment with Ri for 24 hr. (B) Impact of Ri on HUVEC proliferation. (C) Impact of Ri on HUVEC morphology.

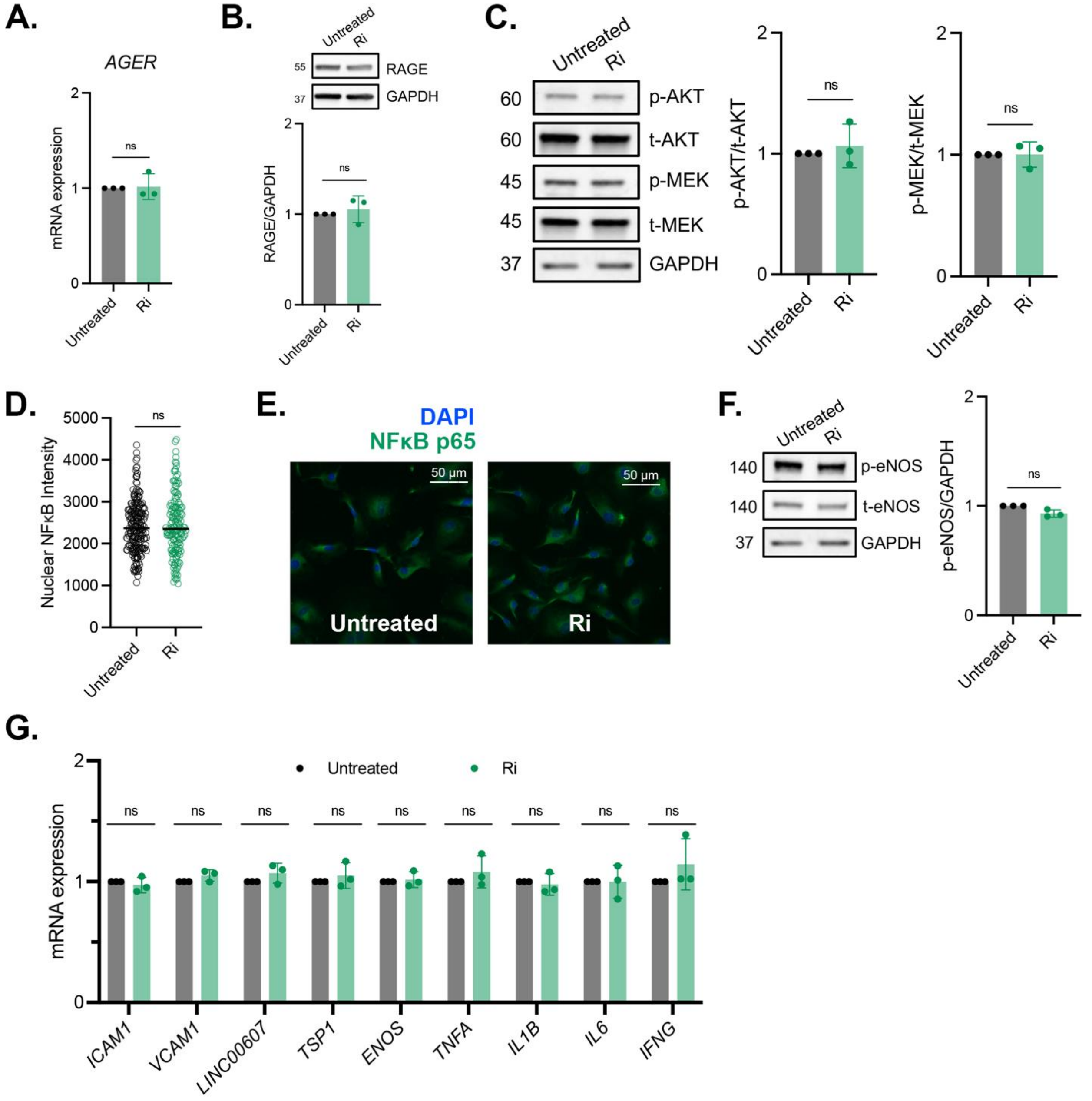

Figure S5. Ri does not impact RAGE expression or activation, or induce endothelial dysfunction. (A) qPCR analysis of *AGER* expression in HUVECs treated with Ri for 1 hr at 100 µM. (B) Western blot analysis of RAGE expression in HUVECs treated with Ri for 1 hr at 100 µM. (C) Western blot analysis of RAGE activation in HUVECs treated with Ri for 1 hr at 100 µM. (D) NFκB nuclear translocation was measured via immunofluorescence and brightfield microscopy. HUVECs were treated with 100 µM Ri for 1 hr. (E) Representative images of (D). (F) Western blot analysis of phosphorylated and total eNOS in HUVECs treated with Ri for 1 hr at 100 µM. (G) qPCR analysis of *ICAM1, VCAM2, LINC00607, TSP1, ENOS, TNFA, IL1B, IL6,* and *IFNG* expression in HUVECs treated with 100 µM Ri for 1 hr.

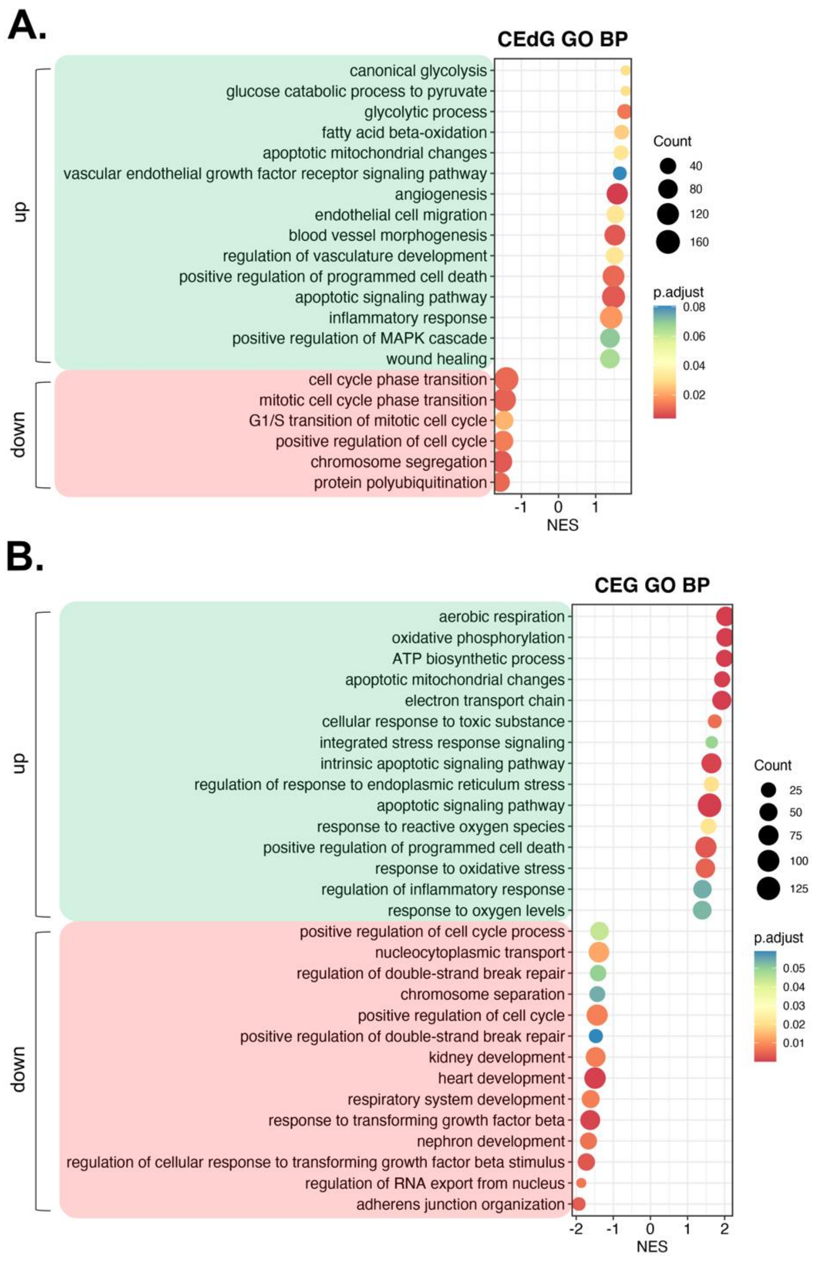

Figure S6. GO-BP pathway analysis of MG-adduct treated HUVECs. HUVECs were treated for 1 hr with 100 ng/mL (A) *R,S*-CEdG or (B) *R,S*-CEG for 1 hr and subjected to RNA sequencing.

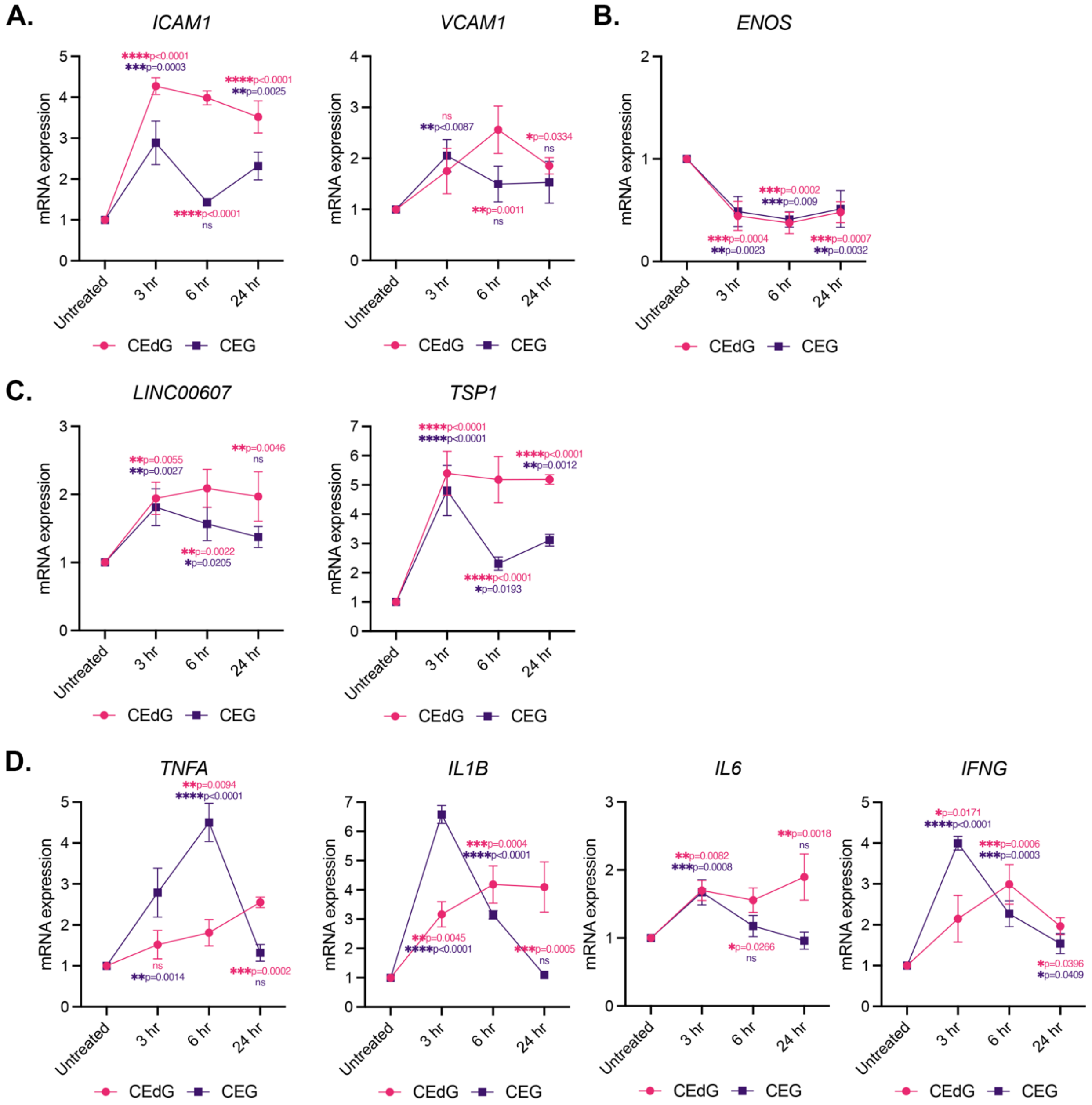

Figure S7. Time dependent effects of MG-adducts on gene expression. qPCR time course analysis of expression of (A) *ICAM1* and *VCAM1*, (B) *ENOS,* (C) *LINC00607*, *TSP1*, (D) and *TNFA, IL1B, IL6,* and *IFNG* in HUVECs treated with 100 ng/mL *R,S*-CEdG or CEG for the indicated timepoints.

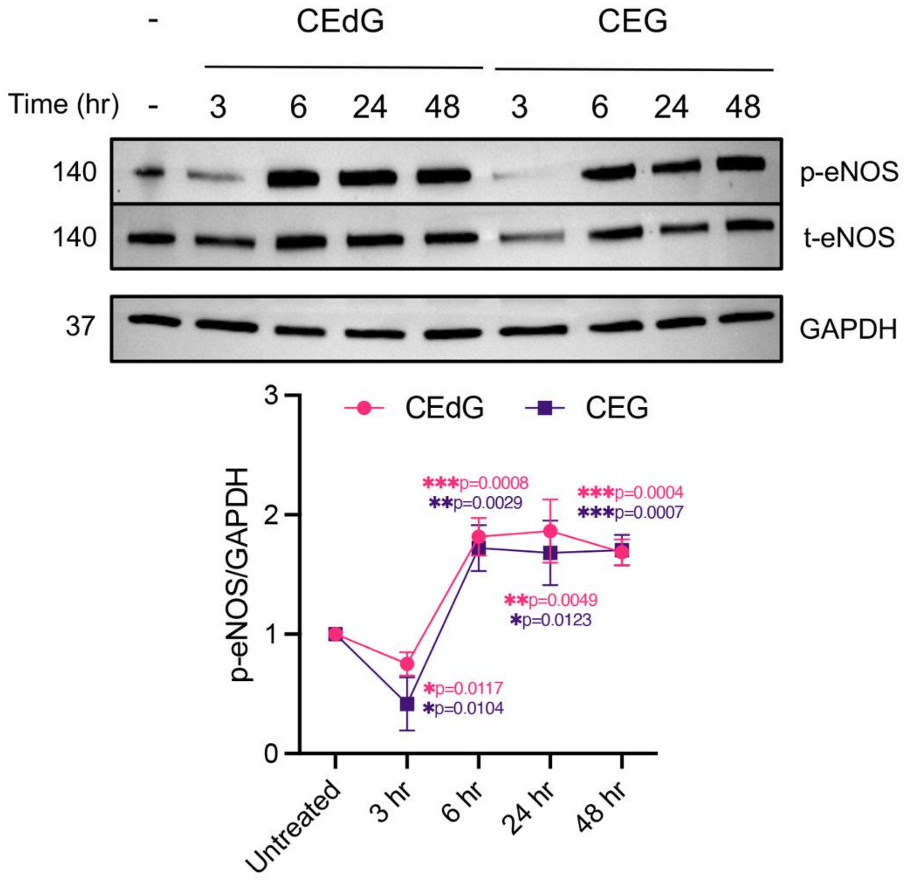

Figure S8. Time dependent effects of MG-adducts on endothelial dysfunction. Western blot time course analysis of expression of total and phosphorylated eNOS in HUVECs treated with 100 ng/mL of *R*,*S*-CEdG or CEG for the indicated timepoints.

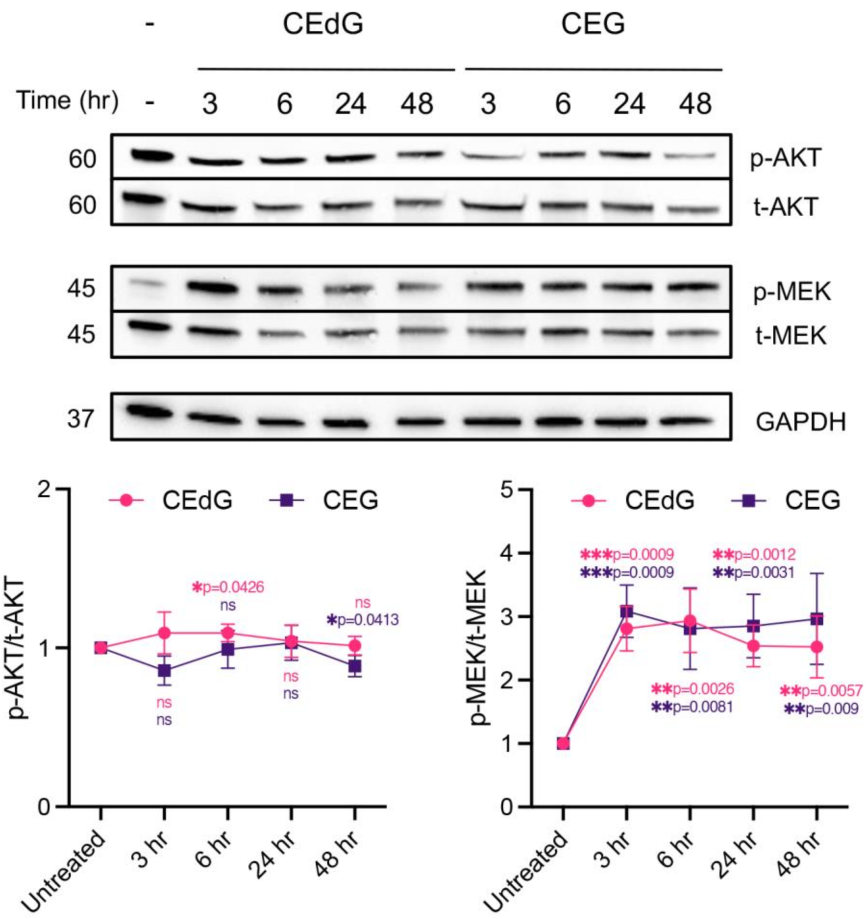

Figure S9. Time dependent effects of MG-adducts on RAGE activation. Western blot time course analysis of expression of total and phosphorylated AKT and MEK in HUVECs treated with 100 ng/mL of *R*,*S*-CEdG or CEG for the indicated timepoints.
